# Evaluating the Quality of Online Patient Information for Prepectoral Breast Reconstruction Using Polyurethane-Coated Breast Implants

**DOI:** 10.1016/j.jpra.2023.10.015

**Published:** 2023-11-03

**Authors:** Edoardo Bruno, Gianluca Borea, Roberto Valeriani, Alessandro De Luca, Federico Lo Torto, Andrea Loreti, Diego Ribuffo

**Affiliations:** a Department of Plastic and Reconstructive Surgery, Sapienza Università di Roma, 00161, Rome, Italy; b Department of Surgical Sciences, Sapienza Università di Roma, 00161, Rome, Italy; c Department of Plastic and Reconstructive Surgery, Azienda Ospedaliera San Giovanni-Addolorata, Via Dell'Amba Aradam 8, 00161, Rome, Italy; d School of Applied Medical-Surgical Sciences, University of Rome Tor Vergata, via Montpellier 1, 00133, Rome, Italy

**Keywords:** Online Information, Polyurethane-Coated Breast Implants, EQIP, Prepectoral Breast Reconstruction

## Abstract

**Background:**

An increasing number of patients are using online information regarding medical issues; however, the Internet is not subject to content ratings or filters. Unreliable information found on the web can heavily influence patients to the extent that it can lead to wrong decisions in the choice of treatment. In our daily experience we meet more informed patients and given the increasing use of polyurethane-coated implants in breast reconstruction in Europe, we wondered about the level of information available online. Our study aims to assess the quality of information available online on breast reconstruction with polyurethane-coated implants.

**Materials and Methods:**

Assuming that the most used search engines are Google and Yahoo, we used a search strategy to identify online information regarding prepectoral breast reconstruction with polyurethane-coated implants. The selected websites were divided into 5 groups (practitioners, hospitals, healthcare portals, professional societies, and encyclopedias), and the quality of information was assessed by using an expanded version of the Ensuring Quality Information for Patients (EQIP) tool, which is a checklist applicable to all existing types of information.

**Results:**

Fifty-six websites were selected and were categorized into 5 groups: 17 practitioners, 9 hospitals, 13 healthcare portals, 7 professional societies, 10 encyclopedias. The average score was 17 points (range: 12 – 25). We found 13 reliable websites with a score higher than 20 using the expanded version of the EQIP tool, whereas 43 were deemed unreliable, as they scored lower.

**Conclusion:**

Proper communication between surgeon and patient is crucial in the therapeutic choice, as the available online information presently is scarce and can lead to wrong decisions if not properly verified.

## Introduction

Breast cancer (BC) is the most common malignancy in women, as reported by the World Health Organization, and its incidence is increasing yearly in most parts of the world.^1^ It is the second leading cause of cancer death among women after lung cancer.^2^ New therapeutic frontiers are moving toward forms of conservative surgery, where 2 types of mastectomies have been described with the added benefit of improved aesthetic results and patient satisfaction, all the while ensuring oncologic radicality: the skin-sparing mastectomy and,^3^ more recently, the nipple-sparing mastectomy.^4^, ^5^, ^6^, ^7^

Currently, immediate breast reconstruction with prosthetic devices is the most popular option after mastectomy,^8^ characterized by excellent aesthetic results and a quick return to daily activities without compromising the quality of life.^9^

Subpectoral breast reconstruction has been performed more often in the past decades; however, its downsides—animation deformity,^10^ muscle spasms, and increased pain—have encouraged surgeons to revisit prepectoral placements.^11^The development of the prepectoral technique in breast reconstruction has allowed surgeons to solve most of these issues. But underneath it, all capsular contracture (CC) remains one of the most common complications after implant placement.

Polyurethane (PU) foam-coating was implemented to silicone implants over 50 years ago, aiming to reduce the risk of CC, which characterized first and second-generation implants with a smooth surface.^11^^,^^12^ Therefore, the use of polyurethane implants has increased in common practice.

The web has increasingly become the main source of information for patients, with nearly half of the European population using it to find health information online; therefore, most patients with BC seek answers to their queries even before consultations with experts.^13^ It is an increasing trend in patients with BC to search for information regarding breast reconstruction and prostheses characteristics online.^14^^,^^15^

However, misinformation and “fake news” are rampant, making evidence-based, reliable information more important than ever as it guides patients in the informed decision-making process. For this reason, we decided to assess the quality of patient information on the characteristics of these prostheses online. Our study aims to precisely evaluate the quality of information published on the PU-coated implants and the breast reconstruction with them.

## Materials and Methods

The study was conceived in August 2023. Assuming that the most popular search engines are Google and Yahoo^16^ and that most users only view the first search pages from the subject of their search query,^17^ we used a search strategy featuring the following terms: ‘‘Polyurethane breast implants”; “Polyurethane-Coated Implant in Breast”; “Polyurethane covered breast implants”; “Polyurethane-coated silicone breast implants”; “Breast reconstruction with polyurethane implants”; “Aesthetic breast surgery with polyurethane implants’’ on Google and Yahoo. The top 50 results were included for each search term. Only websites that met the inclusion criteria were analyzed in the study, thus excluding scientific articles, duplicates, and nonrelevant articles, such as videos or blogs. The selected websites were divided into 5 groups (practitioners, hospitals, healthcare portals, professional societies, and encyclopedias). The quality of information was assessed using the expanded version of the Ensuring Quality Information for Patients (EQIP) tool, a checklist applicable to all existing types of information.^18^^,^^19^ This survey comprises 36 questions, dividing information into 3 sections: content, structure, and identification data.

The first part of the questionnaire focuses on the type of medical intervention, the procedures, and the services offered during hospitalization and postoperative management. It also analyzes benefits and adverse effects and risks, both from a qualitative point of view, such as the reduction of the risk of prosthetic CC, the greater adherence of the prosthesis to the surrounding tissues, the longer duration, or disadvantages, such as greater difficulty in insertion, or the extraction of the same, and the higher cost. From the “quantitative” point of view, we mean to convey data on the same subjects but in the form of numbers with statistical analysis.

The second section analyzes who provides the information, who produces or promotes the documentation, whether the document is updated, and whether there is a bibliography with articles supporting the statements. The last part evaluates whether the text is written clearly, directly, and easily understandable for most users without using specialized or difficult terminology. Each question is assigned the same score of 1, thus obtaining a result between 0 and 36. A score of 20 corresponds to the 75th percentile; therefore, a website with a rating of 20 points or more is considered reliable, whereas a website with a low score is considered unreliable.

## Results

Fifty-six websites were selected and were categorized into 5 groups: 17 practitioners (30%), 9 hospitals (16%), 13 healthcare portals (24%), 7 professional societies (12%), and 10 encyclopedias (18%). All of them underwent qualitative and quantitative assessment using the expanded EQIP tool. (Table 1). The average score was 17 points (range: 12 – 25). We found 13 reliable websites (23.2%), whereas 43 (76.8%) were deemed unreliable. Very few websites talked about potential postoperative warning signs (only 11) and how to manage potential complications (8). Even fewer sites exposed costs or financial aspects of the procedure (only 7). Only 10 websites disclosed other alternative sources of information. Such results are particularly significant when found in websites such as healthcare portals, professional societies websites, and encyclopedias, which respectively averaged a score of 17 (only 30% scoring more than 20), 17.20 (one scoring more than 20) and 15.75 (none more than 20). The hospital websites were the most reliable, reporting an average score of 19.10, with nearly half of the websites exceeding 20 points.

**Table 1:** EQIP tool results applied to the 56 eligible websites about polyurethane breast implants research on Google and Yahoo.

QUESTION	Yes (%)	No (%)
*Content data*		
1. Initial definition of which subjects will be covered	56 (100)	0 (0)
2. Coverage of the above-defined subjects	56 (100)	0 (0)
3. Description of the medical problem	49 (87.50)	7 (12.5)
4. Definition of the purpose of the medical intervention	26 (46.50)	30 (53.50)
5. Description of treatment alternatives (including no treatment)	9 (16)	47 (84)
6. Description of the sequence of the medical procedure	15 (26.78)	41 (73.22)
7. Description of qualitative benefits	44 (78.60)	12 (21.40)
8. Description of quantitative benefits	27 (48.21)	29 (51.79)
9. Description of qualitative risks and side-effects	29 (51.79)	27 (48.21)
10. Description of quantitative risks and side-effects	20 (35.70)	36 (64.30)
11. Addressing quality of life issues	2 (3.50)	54 (96.50)
12. Description of how potential complications will be dealt with	8 (14.28)	48 (85.72)
13. Description of precautions that the patient may take	10 (17.85)	46 (82.15)
14. Mention of alert signs that the patient may detect	11 (19.64)	45 (80.36)
15. Addressing medical intervention cost and insurance issues	7 (12.50)	49 (87.50)
16. Specific contact details for hospital services	9 (16)	47 (84)
17. Specific details of other sources of reliable information/support	10 (17.85)	46 (82.15%)
18. The document covers all relevant issues on the topic	4 (7)	52 (93%)
*Identification data*		
19. Date of issue or revision	53 (94.65)	3 (5.35%)
20. Logo of the issuing body	54 (96.50)	2 (3.50)
21. Name of persons or entities that produced the document	35 (62.50)	21 (37.50)
22. Name of persons or entities that financed the document	4 (7)	52 (93.34%)
23. Short bibliography of evidence-based data used in the document	13 (22.22)	43 (76.7)
24. The document states if and how patients were involved/consulted in its production	2 (3.50)	54 (96.50)
*Structure data*		
25. Use of everyday language. explains complex words or jargon	53 (94.65)	3 (5.35)
26. Use of generic names for all medications or products	46 (82.14)	10 (17.86)
27. Use of short sentences	54 (96.50)	2 (3.50)
28. The document personally addresses the reader	50 (89.28)	6 (10.72)
29. The tone is respectful	54 (96.50)	2 (3.50)
30. Information is clear	51 (91.08)	5 (8.92)
31. Information is balanced between risks and benefits	19 (33.93)	37 (66.07)
32. Information is presented in a logical order	50 (89.28)	6 (10.72)
33. The design and layout are satisfactory	27 (48.21)	29 (51.79)
34. Figures or graphs are clear and relevant	9 (16)	47 (84)
35. The document has a named space for the reader's notes	7 (12.50)	49 (87.50)
36. The document includes a consent form. contrary to recommendations	0 (0)	56 (100)

Analysis of the identification data section showed that most websites presented a date and a logo. Almost 40% of the sites did not disclose who produced the documentation, and 93% did not disclose any source of promotion or financial sponsors. Only 13 sites reported a bibliography of reliable data used in the document. The assessment of data related to the last section of the tool is characterized by a discrepancy in the perception of benefits and risks—although the description is accurate, a lack of graphic sections and images is reported in 9 cases, which could hamper their clear understanding.

## Discussion

Our study was conceived to analyze the quality of information available on the web about breast reconstruction with PU-coated implants, as the request for this type of reconstruction has increased by patients. The information available online on this topic is copious, and the information available on the Internet varies in quality. The analysis of the websites selected in our study showed a lack of online information, especially regarding the description of alternative procedures and the side effects of the same, managing potential complications, identifying red flags, and resolving quality of life and cost issues. Overall, only a few websites were able to expose the problem regarding the use of PU prostheses for breast reconstruction, informing readers correctly about the risks and benefits and, above all, mentioning the alternatives to them.

Hospital portals have collected average EQIP values higher than the websites of operators and professional societies, with all categories lacking reliable references and often even without mentioning who produced or sponsored the document. Hospital portals had higher reliability, with 45% scoring above 20. In comparison, practitioner portal sites and encyclopedias scored significantly less. Additionally, we point out that most evaluated websites lacked information on the topics in the content data section.

None of the websites evaluated in this study met all 36 elements of the modified EQIP tool. The reason can be identified both in the lack of any process of reviewing or management of the information for the sites that are available online and attributed to the fact that major search engines such as Google or Yahoo list the websites on a most viewed basis, rather than listing the findings according to quality and reliability.

For implant-based immediate reconstructions, the devices can be placed both in the submuscular and prepectoral plane with the benefits of prepectoral procedures by sparing the pectoralis major muscle, no functional impairment is caused,^20^ and animation deformity can be avoided as it is caused by the contraction of the muscle over the implant.^21^ Furthermore, it is a procedure with shorter intraoperative times^22^ and improved esthetic outcomes, which is why some surgeons argue that this modality should be considered in any patient candidate for immediate reconstruction.^23^^,^^24^ However, prepectorally placed implants are commonly associated with an increased risk of developing CC.^25^ PU in breast implant coating has been deemed particularly interesting as it has been shown to significantly lower this risk,^26^^,^^27^ compared with smooth prosthesis,^28^ in patients undergoing post mastectomy radiation therapy.^29^ This is likely because PU coating prevents the organized alignment of myofibroblasts, causing CC. After the capsule forms around the implant, the PU foam breaks down to become an integral part of the capsule. In recent years, the use of PU-coated implants has increased in patients undergoing breast augmentation surgery also because it significantly reduces the risk of implant displacement and CC, decreasing the possibility for revision, thus proving a great option for augmentative mammoplasty.^30^

In recent years, thanks to the ever-increasing spread of online news,^31^ patients use the Internet more often as a means to obtain information about reconstructive options after mastectomies and on the types of breast implants, even before hearing an accurate opinion from an expert doctor.^32^ As a result of this trend, practitioners encounter more informed patients who actively participate in their therapeutic choices during consultations,^34,35,36^but the problem arises because of the unreliability and poor quality of the information that patients can find online. Finding and managing high-quality Internet information on breast surgery can be difficult;^33^ the same sites of plastic surgeons should contain more information, analyzing the alternatives to be proposed to patients and exposing more clearly the risks and complications of the procedures and the costs of their services. They should use reliable references to describe their practice on their websites and refer to high-evidence articles published in high-impact factor journals to keep up to date.

However, our study has some limitations: first, only English-language websites were included in our selection,^34^ thus, the quality of websites in different languages remains unknown. This might be relevant as PU-coated websites in breast reconstruction are largely used in the European Union, where the percentage of English speakers is variable.^35^ Furthermore, as the websites shown constantly change their order based on the view counts and we analyzed only the first 50 websites through keyword search, our study is a snapshot from a precise moment in time, which may be subject to changes in the future. Furthermore, the tool used to assess the quality of online information (EQIP) was designed by experts in the field; a substantial effort should be made to involve patients in producing and evaluating medical documents of which they are the recipients. After all, only patients can validate that these are suitable for their information needs. EQIP was developed for use by patient information managers and healthcare professionals and requires at least some knowledge of the topics.^36^

## Conclusion

Our study offers a report on existing information relating to breast reconstruction techniques with PU implants available to BC patients or women who want to undergo cosmetic breast surgery. Although the information available on the web is not subject to a control of the source's reliability, it can influence the patient and his questions to the physician about the personal clinical condition and the therapeutic decisions related to it. The quality of the information available, while better when offered by hospital portals compared to operators, professional societies, and encyclopedias, should be improved: we believe that the Internet should not be used as the main source of medical information, and physicians should maintain the role model, waling these patients through the intricacies of gender-reassignment surgery. Medical information to be published on online portals should be carefully planned, setting up which information to give and how to make it readable. Raising the readability level can make the difference between successful or failed communication, significantly improving the management of breast reconstruction, making patients comprehend the different procedures and better able to choose their reconstructive option.

## Ethical Approval Statement

Not required.

## Declaration of Competing Interest

The authors report no conflict of interest.
